# Identification of gluten-like proteins in selected pod bearing leguminous tree seeds

**DOI:** 10.1371/journal.pone.0249427

**Published:** 2021-04-05

**Authors:** Mostafa Taghvaei, Brennan Smith, Gamze Yazar, Scott Bean, Michael Tilley, Brian Ioerger

**Affiliations:** 1 Animal, Veterinary and Food Sciences, University of Idaho, Moscow, Idaho, United States of America; 2 United States Department of Agriculture, Agricultural Research Service, Southern Regional Research Center, New Orleans, Louisiana, United States of America; 3 United States Department of Agriculture, Agricultural Research Service, Center for Grain and Animal Health Research, Manhattan, Kansas, United States of America; The Chinese University of Hong Kong (Shenzhen), CHINA

## Abstract

The protein composition, molecular weight distribution, and rheological properties of honey locust, mesquite, Kentucky coffee tree, and carob seed germs were compared against wheat gluten. Polymeric and Osborne fractionation protocols were used to assess biochemical properties. Dynamic oscillatory shear tests were performed to evaluate protein functionality. All samples had similar ratios of protein fractions as well as high molecular weight disulfide linked proteins except for the Kentucky coffee tree germ proteins, which were found to have lower molecular weight proteins with little disulfide polymerization. Samples were rich in acidic and polar amino acids (glutamic acid and arginine,). Rheological analyses showed that vital wheat gluten had the most stable network, while Kentucky coffee seed proteins had the weakest. High molecular weight disulfide linked glutenous proteins are a common, but not universal feature of pod bearing leguminous trees.

## Introduction

Glutenous proteins are a rare occurrence and few plants outside of wheat have proteins with demonstrated gluten-like properties. To the authors knowledge, few proteins have been shown to behave similarly as gluten to form a “true dough” that is workable/moldable by hand. These are caroubin from the germ of the carob tree (*Ceratonia siliqua*) [1–5], zein from maize (*Zea mays*) [6–9], and proteins of the marama bean (*Tylosema* species) [10]. The rubber-like physical properties of gluten have been selected for hundreds of years, and gluten was isolated for the first time about 300 years ago [11]. Gluten creates a viscoelastic protein network when combined with water and mixed, which is a key to the desired textural properties of bread and bakery products. The exact mechanism of how such a protein network is developed and how other dough components and ingredients contribute is not fully understood. It is believed that high molecular weight proteins create a network with the help of intermolecular interactions such as hydrogen bonding, disulfide bonding, and ionic interactions [12]. Such interactions enable large polymeric proteins to create three-dimensional networks that form a viscoelastic rubber-like material known as dough. When searching for alternative protein sources to gluten, the ability to form similar networks must be taken into account.

Because leguminous pod bearing trees including both mesquite and carob trees have been shown to have seeds with high protein content, and in some cases functionalities similar to gluten [1–4], greater exploration in this group of plants is a logical next step in finding alternatives to gluten. Mesquite (*Prosopis juliflora*) is a tree belonging to the family of Leguminosae and subfamily of Mimosoideae. This perennial ever-green tree is fast growing, resistant to drought, and usually grows in semi-arid regions of the world [13]. Another leguminous tree native to the Mediterranean region is carob (*Ceratonia siliqua*). The traditional food application of carob has been carob seed gum, also called locust bean gum. The industrial production of carob seed gum leaves behind a large amount of carob germ flour as a by-product, which could be an excellent source for protein extraction [5]. Honeylocust (*Gleditsia triacanthos*) is another tree that has gained interest for food and feed application. Improved varieties of honey locust have a rapid growth rate, produce seeds with high nutritive value, and have a higher seedpod yield [14]. Kentucky Coffee tree *(Gymnocladus dioicus)* is also a pod bearing tree which is native to the central United States and produces reddish brown to black, 12 to 25 cm long pods that contains dark brown seeds [15].

Given the ability of carob germ flour proteins to form dough and similarities to trees with similar life histories, the objective of this study was to analyze the germ protein composition and molecular weight distribution difference among carob, honey locust, mesquite, and Kentucky coffee tree seeds, as well as their rheological properties. This information is important for determining gluten-like functionality and will provide an opportunity to better understand the physicochemical basis of the viscoelastic properties of plant protein complexes such as leguminous tree seeds proteins and wheat gluten. Discovery of glutenous proteins is key for the future development of analogues to wheat bread [5, 16].

## Results

### Total protein

Protein content of all samples was measured via nitrogen combustion using a conversion factor of 6.25. From high to low, the total protein content of mesquite, carob, honey locust, commercial carob, and Kentucky coffee seeds were 63.76, 52.96, 50.65, 49.90, and 31.53% (w/w) respectively. In general, all seed germs had a relatively high protein content.

### Polymeric protein extraction

**Fig 1:**
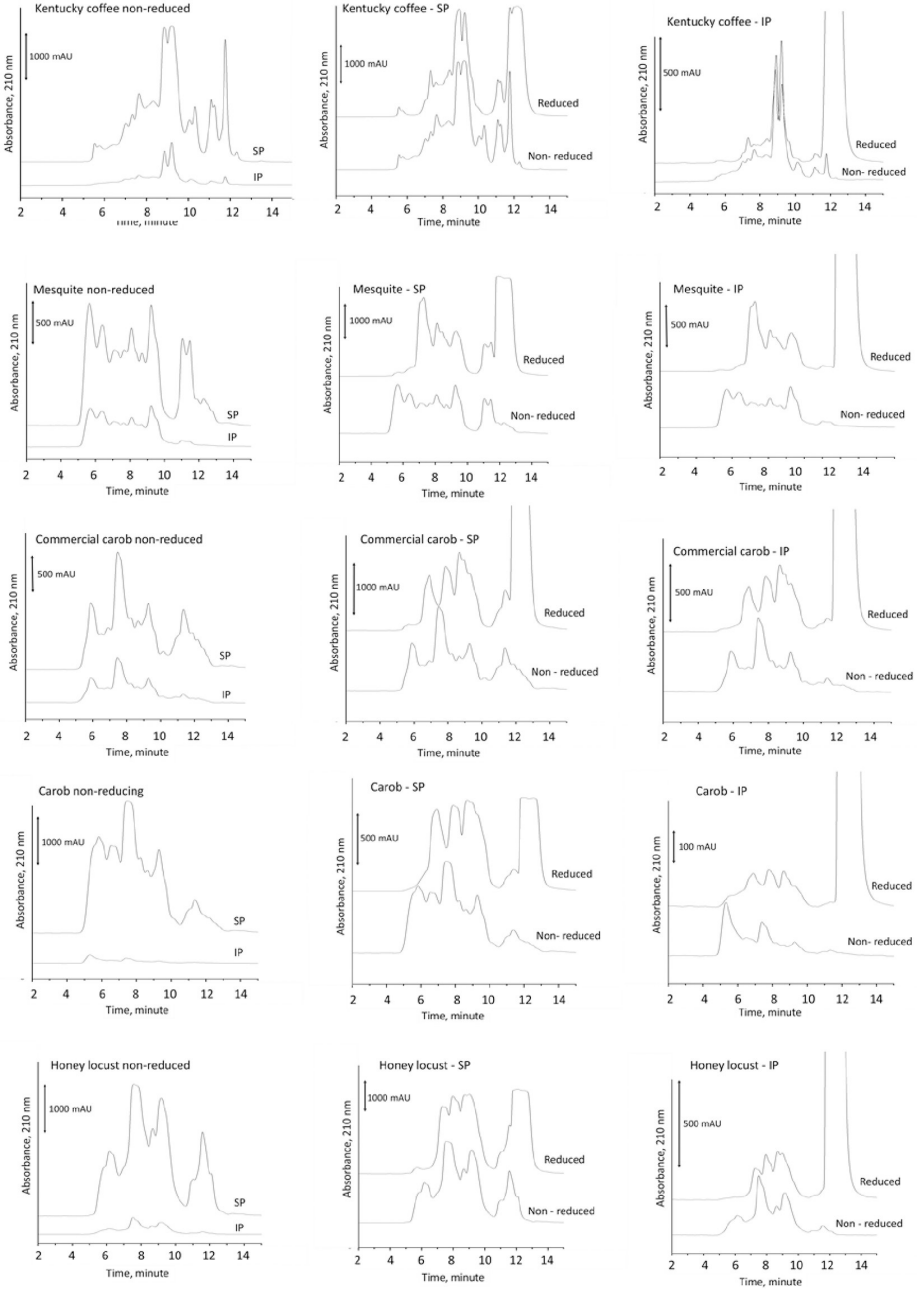
Size exclusion chromatograms obtained from soluble protein (SP) and insoluble protein (IP) fractions in reduced and non-reduced forms.

Size exclusion chromatography separates proteins based on their hydrodynamic radius and provides information on molecular weight distribution of proteins in a sample. Table 1 shows the percentage of the soluble, insoluble, and residue protein fractions of each sample calculated by dividing the total peak area of each fraction by the sum of areas from all fractions. For all samples, soluble proteins are in the highest quantity, followed by insoluble proteins and residue proteins. This is also apparent in Fig 1 where chromatograms obtained from the soluble protein fraction (SP) and the insoluble protein fraction (IP) are compared. A common trait that is observed in all five samples is that soluble protein fractions show a high amount of proteins with low hydrodynamic radius (late eluting peaks) that are absent in insoluble fractions. This suggests that smaller, more readily soluble proteins predominate in all samples. SEC also showed that there is a large amount of variability between samples in their IP molecular weight distribution.

**Table 1:** The size exclusion chromatography of soluble, insoluble, and residue protein fractions from polymeric protein extraction procedure, and Osborne fractions.

Sample name	Polymeric Protein Extraction (% of total peak area)			Osborne fractions (% of total peak area)			
	Soluble proteins	Insoluble proteins	Residue proteins	Albumins/globulins	Prolamins non-reduced	Prolamins reduced	Glutelins
**Carob**	95.7 ± 2.3	3.8 ± 0.3	0.6 ± 0.0	44.6 ± 0.9	8.5 ± 0.1	2.7 ± 0.2	44.2 ± 0.2
**Commercial carob**	69.4 ± 16.6	20.6 ± 5.9	10.1 ± 2.2	64.8 ± 1.4	11.6 ± 0.0	3.7 ± 0.1	19.9 ± 0.1
**Mesquite**	70.6 ± 3.2	19.2 ± 1.1	10.2 ± 0.6	52.3 ± 1.4	8.7 ± 1.2	2.0 ± 0.0	37.0 ± 0.4
**Kentucky coffee**	88.5 ± 4.3	10.1 ± 1.1	1.4 ± 0.2	84.8 ± 1.4	3.9 ± 0.2	1.2 ± 0.1	10.1 ± 0.0
**Honey locust**	92.3 ± 0.1	6.9 ± 0.4	0.8 ± 0.1	76.9 ± 0.7	4.1 ± 0.4	1.8 ± 0.0	17.2 ± 0.7

Comparing the peaks of the reduced with non-reduced fractions revealed a shift to later elution times in all samples, except for the Kentucky coffee tree germ proteins. This shift in peaks is the result of the splitting of more complex proteins into their subunits by disulfide bonds cleavage by 2-ME. From Table 1 and Fig 1, it appears Kentucky coffee seed proteins contain much lower molecular weight proteins with little to no disulfide linked polymers. Note, all reduced samples had a large peak between minutes 12 to 14, which was the 2-ME added to the samples as a reducing agent.

### Osborne fractionation

**Fig 2:**
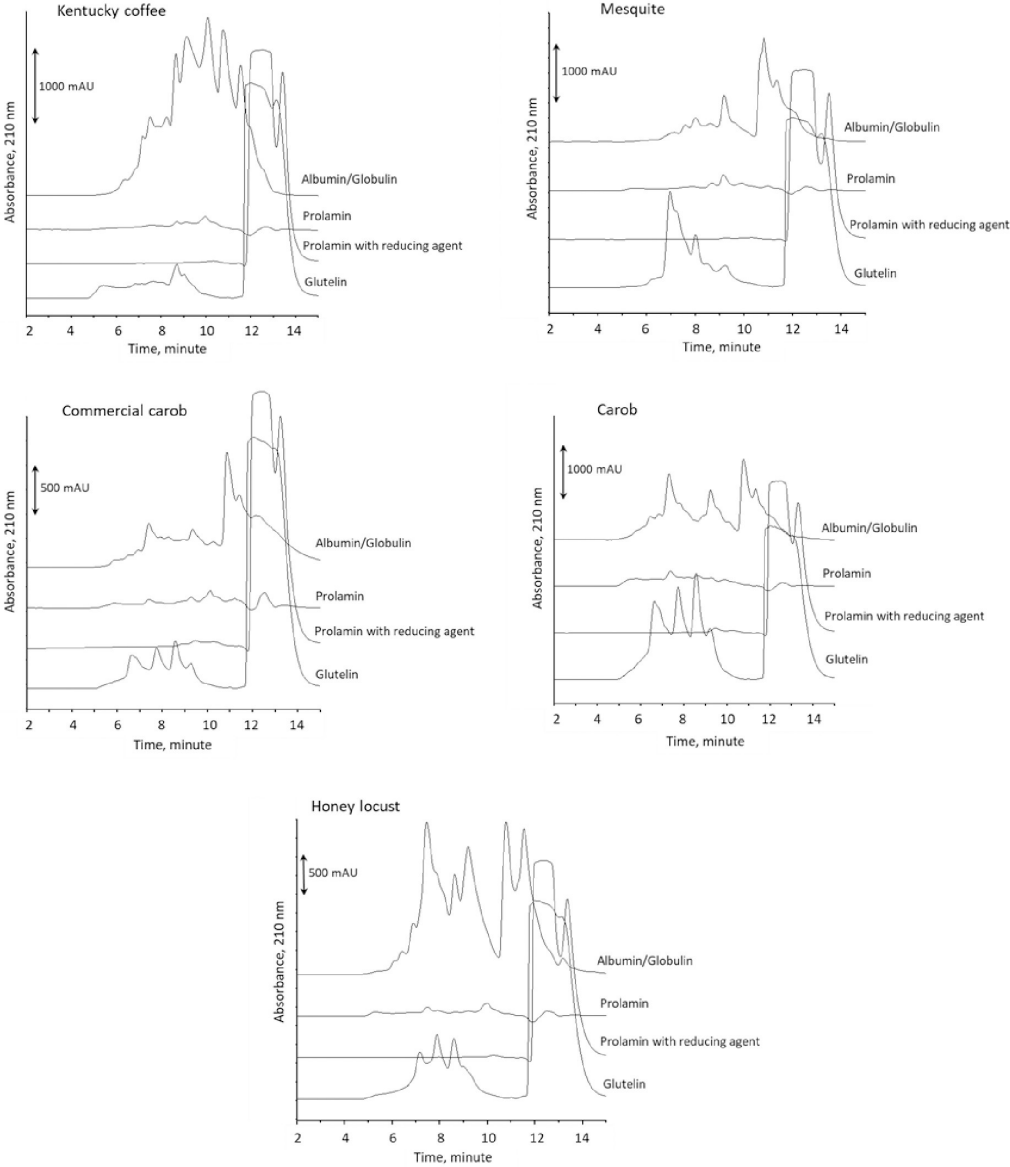
Size exclusion chromatograms obtained from Osborne fractionation analysis.

Based on the Osborne fractionation scheme, proteins were divided into albumin/globulin, prolamin, reduced prolamin, and glutelin fractions [5]. This classification of proteins by solubility is a classical technique for characterizing wheat and similar proteins. Individual protein fractions were separated by size exclusion chromatography (Fig 2). The majority of proteins for all samples were in the albumin/globulin fraction, which has the highest solubility in aqueous salt solutions. The prolamin fraction shows lower amount of proteins, and reduced prolamin fraction shows little to no protein. The glutelin fractions also show a large portion of proteins. In the carob seed sample, the amount of glutelin is the highest, and almost the same as albumin/globulin fraction (Table 1). The Osborne composition of the laboratory produced carob germ flour was again similar to the results by Smith et al. [5]. Mesquite germ flour was similar in composition to the carob samples (Table 1), but with greater amounts of protein.

### Microfluidic separation (lab-on-a-chip)

**Fig 3:**
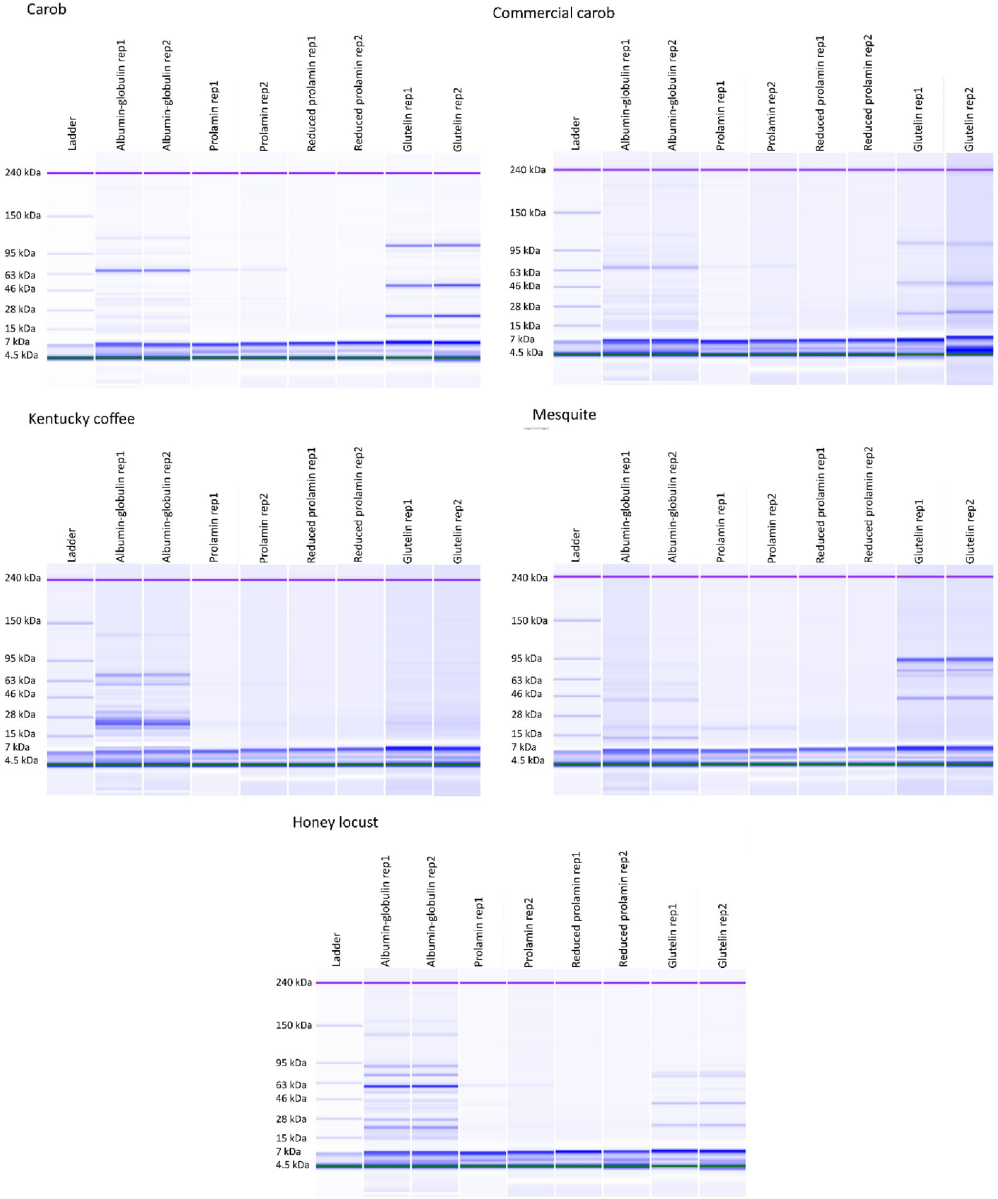
The SDS-PAGE analysis of Osborne fractions.

Fig 3 shows the microfluidic size separation of Osborne fractions from all five samples. Each band represents a protein with a specific molecular weight with the intensity of the color relating to the protein’s concentration. The molecular weights were determined based on a set of standard proteins with molecular weights of 4.5, 7, 15, 28, 46, 63, 95, 150, and 240 kDa. The prolamin fractions (reduced or non-reduced) contained little to no protein. Most of the proteins were observed in the albumin-globulin fraction. This concurs with our results of size exclusion chromatography of the Osborne extracts. The majority of proteins in these fractions show a molecular weight of approximately 28 and 63 kDa. The glutelin fractions also appear to contain 3 major proteins, which was similar to the findings by Smith et al. [5]. Carob and commercial carob samples show the same proteins with commercial carob having the lower color intensity (lower concentration). Honey locust and mesquite also show three major proteins in their glutelin fraction, but with different molecular weights. Regarding the number of proteins, the highest number of bands were observed in the albumin-globulin fractions, which showed great diversity in molecular weight distribution.

### Amino acid analysis

The amino acid composition of all 5 samples were compared to vital wheat gluten in both concentration and ratio to total amino acids (Table 2). As noted in Table 2, glutamic acid and aspartic acid represent, both glutamine plus glutamic acid and asparagine plus aspartic acid respectively. As expected, the major amino acid of vital wheat gluten was glutamic acid (233.2 mg/g) comprising 36.6% of total amino acids [17]. Glutamic acid contains a carboxylic acid group attached to the end of the C3 side chain, which provides ionic interaction sites with divalent ions in the dough to stabilize the viscoelastic structure of gluten [17]. Among samples, carob had the highest percentage of glutamic acid (24.7 and 27.6% for carob and commercial carob, respectively) followed by honey locust and mesquite containing 22.7 and 20.1% glutamic acid, respectively. Glutamic acid was the most predominate amino acid in all five samples, which could to some extent explain the similar structure of those proteins to gluten when mixed with water.

**Table 2:** The amino acid composition of all five samples (concentration and ratio) compared with gluten.

Amino acid	Concentration (mg/g)						Ratio to total amino acids (%)					
	Vital gluten	Carob	Commercial carob	Kentucky coffee	Honey locust	Mesquite	Vital gluten	Carob	Commercial carob	Kentucky coffee	Honey locust	Mesquite
Aspartic acid^1^	21.6	40.0	35.0	24.1	49.3	50.1	3.4	8.6	8.9	10.7	10.2	8.9
Threonine	15.7	14.3	12.8	14.0	14.4	12.5	2.5	3.1	3.3	6.2	3.0	2.2
Serine	36.8	26.4	22.3	17.2	29.0	29.2	5.8	5.7	5.7	7.6	6.0	5.2
Glutamic acid^2^	233.2	115.3	108.4	34.1	109.7	112.6	36.6	24.7	27.6	15.1	22.7	20.1
Glycine	26.4	27.3	23.4	13.2	26.6	31.2	4.2	5.8	6.0	5.9	5.5	5.6
Alanine	18.3	21.4	19.1	10.9	23.7	27.8	2.9	4.6	4.9	4.8	4.9	5.0
Cysteine	32.0	41.4	12.2	8.8	14.1	30.9	5.0	8.9	3.1	3.9	2.9	5.5
Valine	13.1	9.0	7.8	5.9	11.9	16.3	2.1	1.9	2.0	2.6	2.5	2.9
Methionine	1.2	3.0	0.9	0.7	1.4	3.7	0.2	0.6	0.2	0.3	0.3	0.7
Isoleucine	10.9	6.9	6.2	4.5	9.2	8.4	1.7	1.5	1.6	2.0	1.9	1.5
Leucine	40.8	27.6	23.2	14.3	32.6	39.8	6.4	5.9	5.9	6.3	6.7	7.1
Tyrosine	22.2	13.3	11.9	7.4	15.3	17.3	3.5	2.8	3.0	3.3	3.2	3.1
Phenylalanine	36.3	16.2	13.1	10.5	19.7	25.5	5.7	3.5	3.3	4.6	4.1	4.6
Lysine	8.4	25.8	22.0	15.9	27.3	21.3	1.3	5.5	5.6	7.0	5.6	3.8
Histidine	11.6	11.0	9.5	5.4	12.6	16.9	1.8	2.4	2.4	2.4	2.6	3.0
Arginine	20.6	60.7	50.9	28.6	66.5	82.3	3.2	13.0	13.0	12.7	13.7	14.7
Proline	87.6	7.1	13.7	10.3	20.4	34.7	13.8	1.5	3.5	4.6	4.2	6.2

^1^ Aspartic acid + asparagine

^2^ Glutamic acid + glutamine

The second most abundant amino acid in gluten was proline at 87.6 mg/g comprising 13.8% of the total amino acids [17]. This high amount of proline and other non-polar amino acids could provide hydrophobic interactions inside protein chains when mixed with water. However, the amount of proline in the tree seed germ samples was not as high as it was in gluten. Levels of other non-polar amino acids (such as glycine and valine) in all five samples was comparable to that of gluten (Table 2).

All samples also showed a high amount of cysteine (highest in carob at 41.4 mg/g). This unique amino acid, which provides disulfide bonding sites to other cysteine groups, is known to have a key role in the viscoelastic structure of gluten. The high amount of cysteine in carob and mesquite provides the possibility of disulfide interactions in those proteins when making dough [5].

### Water hydration capacity

Water hydration capacity (WHC) is an important functional property for an ingredient used in food formulations where there is interaction with water. The WHCs of samples in increasing order were 1.62±0.02, 1.73±0.05, 1.79±0.06, 1.81±0.02, 2.04±0.03, and 2.32±0.06, for Kentucky coffee, vital gluten, commercial carob, mesquite, honey locust, and carob, respectively. Except for Kentucky coffee, all the other samples had a WHC higher than gluten. The laboratory made carob germ flour showed the highest WHC with a hydration of 2.32 mL/g. The value for gluten was 1.73 mL water per gram gluten. This shows the superiority of carob and honey locust over gluten for hydration in food formulations.

### Dynamic oscillatory shear analysis

**Fig 4:**
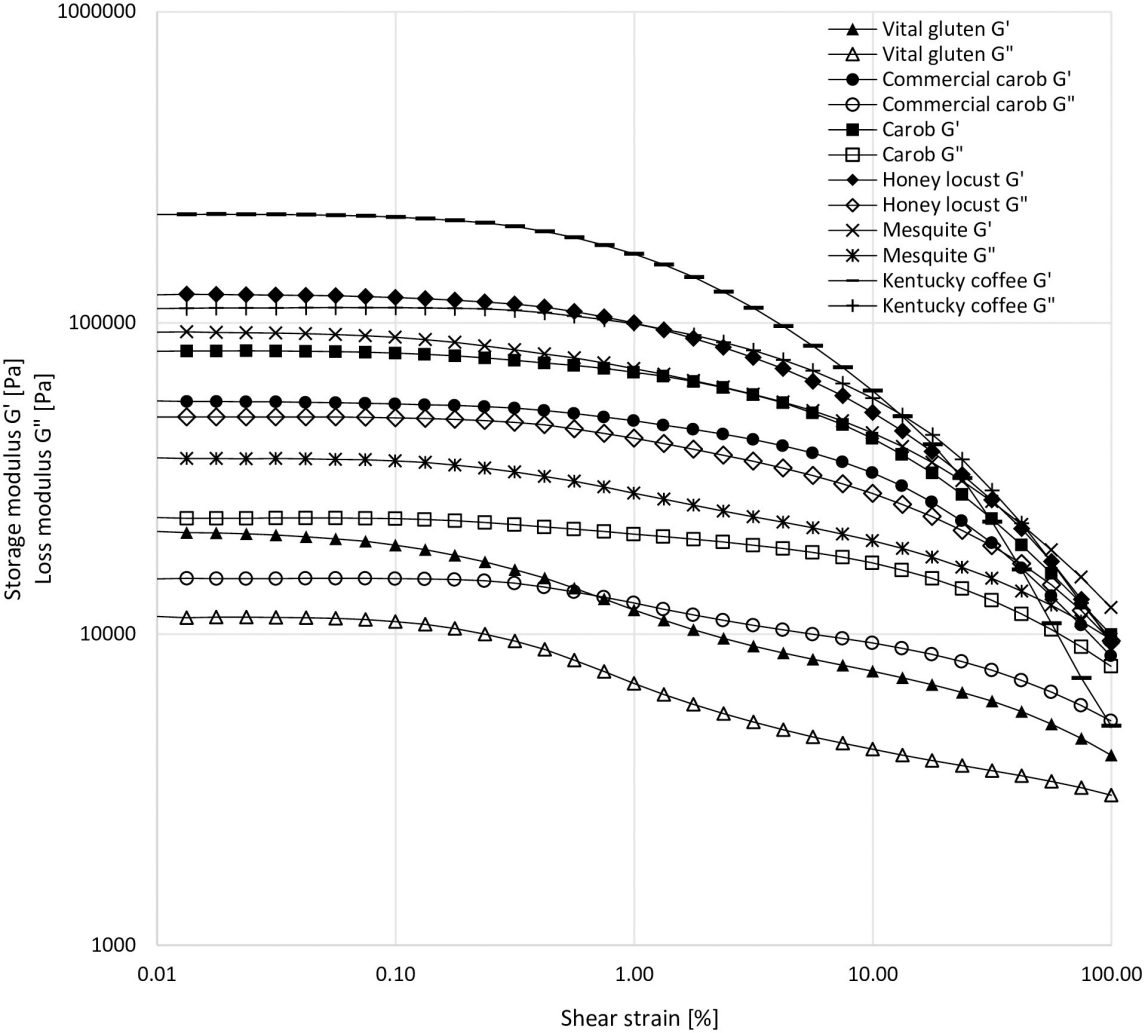
Strain sweep data for the protein samples (ω = 1 Hz, T = 20°C).

The data in the linear viscoelastic region showed that Kentucky coffee germ had the highest elasticity among all protein samples (Fig 4). Gʹ values were ~2x10^5^ Pa for the Kentucky coffee seed germ at low applied strains, whereas vital wheat gluten showed the lowest Gʹ values with 2x10^4^ Pa, showing a high degree of variability in Gʹ with one order of magnitude difference between the highest and lowest values. The honey locust sample was found to be the only protein showing a crossover at a strain value of around 20%. Gʹ and Gʹʹ for the honey locust protein represented a crossover at the highest strain applied (100%). However, Gʹ and Gʹʹ values for the rest of the proteins did not show a crossover up to the highest strain applied. The crossover observed for the Gʹ and Gʹʹ values of the Kentucky coffee and honey locust showed the highest elastically dominated linear rheological properties, indicating a structural decay, which resulted in a more viscously dominated behavior for these proteins as the amplitude of strain increased.

The laboratory produced carob germ flour represented higher Gʹ and Gʹʹ values compared to the commercial carob. Gʹ values for the carob protein overlapped with the Gʹ values of mesquite. However, the magnitude between the Gʹ and Gʹʹ values for the carob protein was higher compared to that of mesquite, indicating a more elastic structure for the carob protein. Commercial carob protein and vital wheat gluten showed similar Gʹʹ profiles throughout the applied strain range.

Frequency sweep tests, where the effect of frequency on the linear viscoelastic properties of different protein doughs were studied, revealed that Gʹ values were higher than Gʹʹ values for all samples within the applied frequency range (0.1–100 rad/sec). At the lowest frequency applied, Gʹ and Gʹʹ values for vital wheat gluten were almost the same. As the frequency increased, this difference started to increase until the frequency reached 0.25 rad/sec. Above this frequency, Gʹʹ values were recorded to be 2 times below Gʹ values.

**Fig 5:**
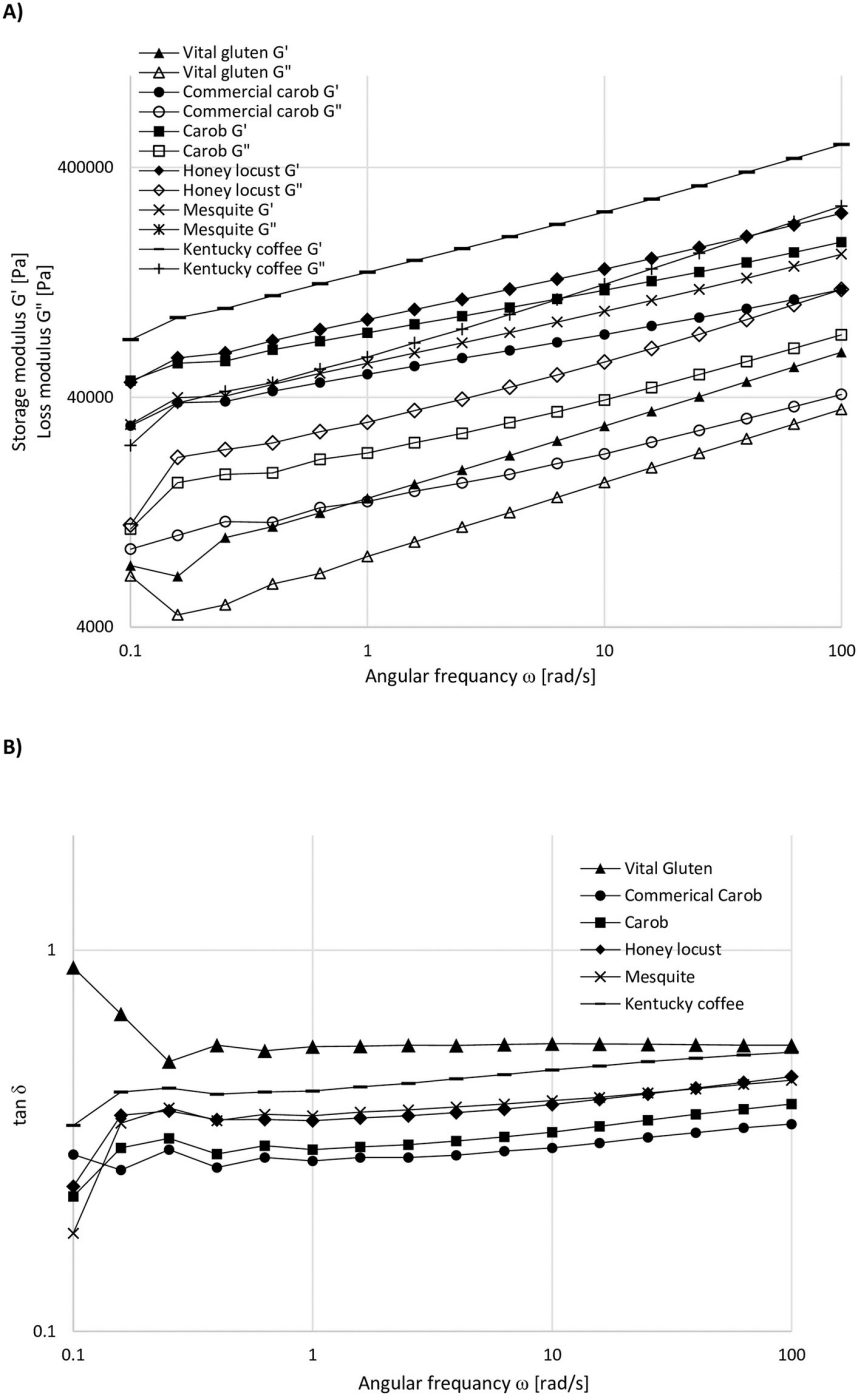
A) Frequency sweep data for the protein samples (γo = 0.02%, T = 20°C), B) Loss factor (tanδ) as a function of frequency for the protein samples.

The ratio of viscous (Gʹʹ) to elastic (Gʹ) components which is known as loss factor (tanδ) is another commonly used parameter to evaluate the dynamic viscoelastic properties of materials [18]. Since Gʹ values are higher than Gʹʹ values for all samples throughout the applied frequency range, tanδ for all samples were lower than 1. All protein dispersions, except for vital wheat gluten, showed increasing tanδ values with respect to increasing frequencies (Fig 5B) suggesting a viscously dominated linear viscoelastic behavior, which is typical for biopolymer gels [19]. Vital wheat gluten represented decreasing tanδ at low frequencies up to 0.25 rad/sec and above this frequency showed a slight increase, then remained consistent at frequencies above 10 rad/sec.

Phase angle δ = 0° or tanδ = 0 corresponds to an elastic response, while δ = 90° or tanδ = ∞ represents a viscous response. The material behavior is described as viscoelastic in the case of phase angle being within the limits of 0°<δ<90° [20, 21]. As pointed out with the strain sweep (Fig 4), frequency sweep (Fig 5A) data and tanδ values (Fig 5B), vital wheat gluten represented the highest viscously dominated viscoelastic behavior with the highest δ values for all frequencies studied. Again, the most consistent δ values observed for vital wheat gluten against the increasing frequency proved the stability of the gluten network. Depending on the phase angle values recorded at frequencies above 1 rad/sec (Table 3), the protein samples are listed as follows in terms of the degree of elasticity dominating their viscoelastic behavior: commercial carob>carob>mesquite≥honey locust>Kentucky coffee>vital wheat gluten.

**Table 3:** Phase angle (δ) values obtained at certain frequencies for the protein dispersions.

*ω* (rad/sec)	δ (°)					
	Vital gluten	Commercial carob	Carob	Honey locust	Mesquite	Kentucky coffee
0.1	42.38	14.94	11.49	18.31	11.69	17.7
1	28.51	15.34	16.78	20.22	20.08	22.7
10	29.61	16.62	18.48	21.91	21.8	25.4
100	29.91	19.25	21.4	25.03	24.25	27.9

## Discussion

The polymeric protein extraction is a biochemical method used to gage wheat gluten quality. Here proteins are sequentially separated unreduced into SP (soluble in SDS), IP (soluble in SDS with mild reduction through ultrasonication), and RP (soluble in SDS with a chemical reductant). For glutenous proteins, greater proportions of IP are indicative of greater quantities of high molecular weight proteins, which can result in increased dough strength and quality [5, 22–24]. While IP is known to lead to improved gluten strength and dough quality in wheat, the proteins in wheat IP fractions are typically skewed to a higher molecular weight [5, 22–24] than what was observed in the experimental tree seed samples. In the tree seed samples, the molecular weight distribution varied widely amongst samples. The Kentucky coffee trees seeds had almost no proteins eluting at the early eluting exclusion peak, while mesquite and the commercially produced carob germ flour had the most (Fig 1). It was also interesting that the molecular weight distribution of the carob samples IP varied within this study and from the work by Smith et al. [5]. The laboratory produced carob germ flour proteins in this project were similar to the data reported in Smith et al. [5]. This suggests that there is a potential for regional, seasonal, and/or genetic influence on the protein composition and quality. Furthermore, disulfide linked high molecular proteins are a key factor in achieving glutenous behavior of proteins [24]. From the microfluidics data, it is evident that proteins from each of the seeds studied varied substantially. This is discernible by the presence and absence of bands from one seed to the next for a given protein fraction. While each of the seeds with glutenous properties had high molecular weight disulfide linked insoluble proteins, it is evident that the composition of these high molecular weight proteins varied. As demonstrated by these data, there are many different routes to achieved gluten-like properties. One of the key parameters for achieving this, seems to be high molecular weight disulfide linked proteins capable of spontaneously forming protein networks in the presence of water. Aside from biochemical aspects, rheological properties are another important factor to understand for application of proteins in the production of wheat-like dough. The G’, G”, and crossover values of this study demonstrated an elastically dominated system, which fits with the polymeric protein extraction and Osborne fractionation data. This is particularly evident in the mesquite and lab produced carob samples, where the lab made carob germ flour and mesquite germ flour were found to be in similar ratios for both extraction techniques (Table 1 & Fig 1). It is also logical that these samples were dominated by elastic properties when compared to wheat since they have low prolamin content, the protein fraction known to provide extensibility to wheat gluten [5].

For frequency sweep tests, as the frequency increased, the difference between the Gʹ and Gʹʹ values became larger (Fig 5), suggesting a less solid-like behavior for all protein samples, except for vital wheat gluten. Furthermore, the consistent ratio between Gʹ and Gʹʹ values with respect to increasing frequencies revealed a relatively stable network for vital wheat gluten compared to the other protein samples. This was likely due to the lack of prolamins in the tree seeds samples, which are predominately gliadins and glutenins in wheat, which aide in cohesiveness of the system [25].

Kentucky coffee germ showed the highest Gʹ and Gʹʹ values for the whole frequency range, while vital wheat gluten represented the lowest Gʹ and Gʹʹ values which concurs with the data obtained through the strain sweep tests in the linear region. At low frequencies, carob and honey locust represented similar Gʹ values; however, the relatively higher Gʹʹ observed for honey locust demonstrated a more viscously dominated viscoelastic behavior. Mesquite, carob, and commercial carob samples exhibited the most elastic behavior at low frequencies due to having the largest difference between Gʹ and Gʹʹ values. This might be explained by the presence of disulfide linked IP polymers and the lack of prolamins. Increasing frequencies resulted in a greater increase in the viscous components of mesquite and carob samples, which made the commercial carob sample the most elastically dominated protein sample among the proteins studied.

The information obtained through tanδ values concurs with the frequency sweep results (Fig 5A). indicating vital wheat gluten had the lowest elastically dominated linear viscoelastic behavior, while having the most stable network among the other protein samples against the applied frequency range. Even though, Kentucky coffee tree proteins showed the highest Gʹ values within the applied strain and frequency ranges, its viscous component (Gʹʹ) showed the highest values among the protein samples tested. Kentucky coffee tree germ also represented a crossover point (Gʹʹ> Gʹ) as the amplitude of strain increased. The dominance of the viscous component in the rheological behavior of Kentucky coffee tree samples resulted in high tanδ values in comparison with the other protein samples studied, suggesting that Kentucky coffee has the highest elastic properties with a weaker network structure that was affected by the applied strain and frequency the most. This was also supported by the biochemical data (Table 1 & Fig 1), demonstrating low quantities of high molecular weight proteins, little disulfide cross-linking, and little prolamin and glutelin in the Kentucky coffee germ samples. Commercial and laboratory carob germ showed similar tanδ trends and had the lowest range of tanδ, suggesting a more rigid structure. Locust and mesquite proteins showed similar behavior for most of the applied frequency range. However, increasing frequency caused a sharper increase in tanδ for mesquite, which means it behaved more elastic at lower frequencies, but the viscous component became more dominant against increasing frequencies compared to honey locust protein. This was further supported by a larger increase in the phase angle values for mesquite compared to that of the honey locust as the frequency increased (Table 3).

In conclusion, most proteins were found in the soluble and albumin-globulin fractions with disulfide cross linkage and molecular weights around 28 and 63 kDa. Samples were rich in acidic and polar amino acids, such as glutamic acid, arginine. Vital wheat gluten showed the lowest elastically dominated linear viscoelastic behavior, while showing the most stable network among the other protein samples. Kentucky coffee tree seed proteins showed the highest elastic properties with a weaker network structure that was affected by the applied strain and frequency the most. From this, it is apparent that glutenous proteins are a common feature in pod bearing leguminous trees. However, results from the Kentucky coffee tree demonstrate that this feature is not universal among trees of this type. Information on the composition, molecular weight distribution, and rheological properties of carob, honey locust, mesquite, and Kentucky coffee tree seeds’ protein fractions, provides vital information for the discovery and potential applications of glutenous proteins in food systems.

## Materials and methods

### Materials

Mesquite of the variety Algarrobo, Carob, and Kentucky coffee tree seeds were obtained from Sheffield’s Seed Co., Inc (Locke, NY, USA). Honey locust seeds were collected locally in the area of Moscow, ID, USA during the 2017 growing year. A commercially available carob germ flour under the market name Grindsted Veg Pro Carob Protein was obtained from Danisco and was used a standard for comparison. Vital wheat gluten (GluVital^™^) was obtained from Cargill (Wayzata, MN).

### Sample preparation

All seeds were removed from the pod and the testa was removed with a 60°C 9.2 M sulfuric acid solution. The sulfuric acid was used to carbonize the testa as described by Battle and Tous [26]. All seeds were treated with sulfuric for 5 hours, except for the Kentucky coffee tree seeds. After treatment with sulfuric acid, the carbonized testa was removed with a water rinse. The Kentucky coffee tree seeds were quite robust and had a 3–4 mm thick testa layer, which took 22 hours to carbonize. The testa removal was optimized so that no endosperm or germ was carbonized. The endosperms were easily removed by hand, leaving only the cotyledons (germ). The germ was then ground into a flour with a lab scale coffee grinder (Krups model F203, China) for 1 minute. For analyses, rheological properties tests were done in triplicates. All other analyses were carried out in duplicates.

### Polymeric protein extraction

Proteins of the various seed germs were extracted by the polymeric protein extraction protocol described by Smith et al. [5]. Proteins were sequentially extracted into soluble proteins (SP) and insoluble proteins (IP). To accomplish this, 20 mg of seed flour was extracted twice with 15 minutes of continuous vortexing in 1 mL of 50mM sodium phosphate, pH 7.0 buffer containing 1% SDS (w/v) to obtain the SP fraction. This extract was collected after 5 min of centrifugation at 9300 x g. The supernatants of the two SP extracts were pooled in a 1:1 ratio. The IP extraction was carried out with 1 mL of the same extraction buffer as the SP extraction, but with the addition of sonication for 30 s at 10 W. This was repeated twice, and extracts were pooled in a 1:1 ratio after centrifuging for 5 min at 9300 x g. To determine how much protein was remaining, the residue proteins (RP) were extracted twice with the same buffer as the SP extraction, but with the addition of 2% 2-mercaptoethanol (2-ME) (v/v) and pooled as above.

### Osborne fractionation

A modified Osborne fractionation was completed as described by Smith et al. [5]. Briefly, proteins were sequentially extracted into four protein classes based on solubility. In order of extraction, the fractions were albumins/glubulins, prolamins, reduced prolamins, and glutelins. The first faction was completed to extract both the albumin and globular proteins using 20 mg of seed flour and a 50 mM Tris-HCl pH 7.8 buffer containing 100 mM KCl and 4 mM EDTA extraction solution. The prolamin fraction was extracted with 50% n-propanol. The reduced prolamin was carried out with 50% n-propanol containing 2% 2-ME (v/v), and the glutelin fractions was completed with a pH 10.0 12.5 mM sodium borate buffer containing 2% SDS (w/v) and 2% 2-ME (v/v). Each extraction used 1 mL of extraction solvent and was carried out with 15 minutes of continuous vortexing, followed by centrifugation for 5 min at 9300 x g. Each fraction was extracted twice and pooled in a 1:1 ratio.

### Protein quantifications

Quantification of total protein was carried out via nitrogen combustion according to AACCI method 46–30.01 [27] using a LECO 628 Nitrogen Determinator (LECO, St. Joseph, MI). A conversion factor of 6.25 was used to convert percent nitrogen to percent protein.

### Size exclusion chromatography

An Agilent 1100 Series HPLC system equipped with an auto-sampler (model G1313A) and a UV detector at 210 nm was used. The separation was carried out on a 30 x 7.8 mm (L x D) BioSep s4000 column (Phenomenex Inc. USA) with exclusion range of 15,000–500,000 Da (0.5% SDS) and using 1% SDS pH 7.0 mobile phase at the flow rate of 1 mL/min. The OpenLAB CDS (ChemStation Edition) software was used to analyze, integrate, and collect data.

### Microfluidic analysis

Microfluidic analysis was performed on the Osborne and polymeric protein extractions on an Agilent Bioanalyzer 2100 (Agilent, Santa Clara, CA). Extracted samples were analysed reduced and unreduced. Analysis was completed in accordance to the instrument manufacturer’s specifications. For reduced samples, 2-ME was used as the reducing agent and added at a rate of 2% (v/v).

### Amino acid analysis

For all samples, 19 common amino acids were determined by HPLC using an Agilent 1100 equipped with a diode array detector. Hydrolysis and amino acid analysis followed methods described by Yufei [28] which used updated protocols first described in the Hewlett Packard Amino Quant Operator’s Handbook [29]. Cysteine was assessed by the creation of Cysteine- 3-mercaptopropionic acid (Cys-MPA) complexes as described by the Amino Quant Operator’s Handbook [29]. This was done to limit the degradation of cysteine during hydrolysis, which was an issue during preliminary optimization. Signal to noise ratios of 10:1 and 3:1 defined the LOQ and LOD, respectively.

### Water hydration capacity

The water hydration capacity of samples was measured according to AACC method [30] number 56–37.01, which determines that amount of water a 1 g of sample can retain under low speed centrifugation. Only enough distilled water is added to saturate the sample without producing a liquid phase. Water hydration capacity was expressed as grams of water retained per gram of sample.

### Dynamic oscillatory shear analysis

Dynamic oscillatory shear tests were conducted using Physica MCR 301 rheometer (Anton Paar, Germany) to study the linear rheological properties of the protein samples Strain sweeps in the strain range of 0.01–100% using the frequency of 1 Hz were conducted to determine the linear region ranges for the samples. Frequency sweep tests were carried out using the frequency range of 0.1–100 rad/sec at a strain value of 0.02% selected in the linear region for each protein sample. All rheological measurements were carried out in triplicate at 20°C. A 25 mm parallel plate geometry and a gap of 2 mm were used. Samples were rested prior to measurement for about 15 minutes until the axial force value decreased to 0.1 N. Samples were coated with vacuum grease in order to prevent moisture loss during the measurements. The average data was plotted using Microsoft Excel (Microsoft Office 365 ProPlus).

## Supporting information

S1 Data (XLSX)

S2 Data (XLSX)

S3 Data (XLSX)

S4 Data (XLSX)

S5 Data (XLSX)
